# Fecal Aliquot Straw Technique (FAST) allows for easy and reproducible subsampling: assessing interpersonal variation in trimethylamine-*N*-oxide (TMAO) accumulation

**DOI:** 10.1186/s40168-018-0458-8

**Published:** 2018-05-18

**Authors:** Kymberleigh A. Romano, Kimberly A. Dill-McFarland, Kazuyuki Kasahara, Robert L. Kerby, Eugenio I. Vivas, Daniel Amador-Noguez, Pamela Herd, Federico E. Rey

**Affiliations:** 1 Department of Bacteriology, University of Wisconsin-Madison, Madison, WI 53706 USA; 2 Center for the Demography of Health and Aging, University of Wisconsin-Madison, Madison, WI 53706 USA; 3 Department of Sociology, University of Wisconsin-Madison, Madison, WI 53706 USA; 4 Present address: Department of Cellular and Molecular Medicine, Cleveland Clinic, Cleveland, OH 44195 USA; 5 Present address: Department of Microbiology and Immunology, University of British Columbia, Vancouver, BC V6T 1Z3 Canada

**Keywords:** Microbiome, Fecal Aliquot Straw Technique, FAST, TMAO, Choline

## Abstract

**Background:**

Convenient, reproducible, and rapid preservation of unique biological specimens is pivotal to their use in microbiome analyses. As an increasing number of human studies incorporate the gut microbiome in their design, there is a high demand for streamlined sample collection and storage methods that are amenable to different settings and experimental needs. While several commercial kits address collection/shipping needs for sequence-based studies, these methods do not preserve samples properly for studies that require viable microbes.

**Results:**

We describe the Fecal Aliquot Straw Technique (FAST) of fecal sample processing for storage and subsampling. This method uses a straw to collect fecal material from samples recently voided or preserved at low temperature but not frozen (i.e., 4 °C). Different straw aliquots collected from the same sample yielded highly reproducible communities as disclosed by 16S rRNA gene sequencing; operational taxonomic units that were lost, or gained, between the two aliquots represented very low-abundance taxa (i.e., < 0.3% of the community). FAST-processed samples inoculated into germ-free animals resulted in gut communities that retained on average ~ 80% of the donor’s bacterial community. Assessment of choline metabolism and trimethylamine-*N*-oxide accumulation in transplanted mice suggests large interpersonal variation.

**Conclusions:**

Overall, FAST allows for repetitive subsampling without thawing of the specimens and requires minimal supplies and storage space, making it convenient to utilize both in the lab and in the field. FAST has the potential to advance microbiome research through easy, reproducible sample processing.

**Electronic supplementary material:**

The online version of this article (10.1186/s40168-018-0458-8) contains supplementary material, which is available to authorized users.

## Background

The human intestine harbors diverse and dynamic microbial communities encompassing species from the three domains of life [1, 2]. These microbial communities encode metabolic functions that complement the human genome and play key roles in our biology and health. While many of these functions are shared among communities from different individuals, large interpersonal differences have been reported [3]. Identifying the consequences of this variation as it relates to host immune responses, drug effectiveness, and metabolism is key to fully understand how microbes modulate our biology and for the successful implementation of precision medicine strategies.

DNA sequencing approaches, such as 16S rRNA gene profiling or shotgun metagenomics, which have dominated the field in the last decade [4], have been instrumental in illuminating the degree of interpersonal variation in gut communities. These methods, however, provide limited insights into the metabolic capabilities of microbes, or how members in the community interact with each other and the host. Germ-free mice colonized with synthetic microbial mixtures provide a complementary platform for dissecting the role(s) of single species or a specific microbial metabolic pathway on host biology. However, most studies taking this approach use constructed communities of low diversity [5–7], in which colonizing species are represented at higher levels than naturally found in the host. While highly tractable, this reductionist approach may overestimate the impact a microbe/pathway has on host biology; or fail to reveal microbial interactions that occur in conditions of high microbial complexity or that may only occur between species that have co-evolved within a specific host. Thus, it is important to validate synthetic community findings with naturally occurring complex communities.

Currently, our ability to move towards larger model communities is limited by the ability to grow many microbes in parallel [8, 9]. One alternative to this limitation is to use uncultured non-defined microbial communities to colonize germ-free mice (i.e., fecal transplants). This approach has been extremely useful in inferring the causal role of microbial dysbiosis with many host phenotypes (e.g., adiposity, insulin resistance) [9, 10]. Key to the success of this approach is the ability to collect samples that can be stored long-term and reproducibly subsampled without compromising cell viability. Currently, this is a laborious process that can be done effectively only on a relatively small scale. Moving to larger studies presents several challenges including shipping/storage/subsampling of material in ways that preserve cell viability and allows for reproducible and repetitive testing of samples.

Current methods of fecal collection and processing for purposes beyond DNA sequencing are cumbersome, messy, and inefficient for handling large number of samples [11–15]. For example, a standard procedure for aliquoting fecal samples entails scooping small amounts of the specimens into multiple 2-ml screw-cap tubes that have been previously labeled. These aliquots are typically for a single use, thus many of them need to be prepared from each specimen (e.g., 20 aliquots), and significant amounts of freezer space is needed for their storage. Importantly, the process of aliquoting samples this way exposes a large fraction of the microbes to air, which decreases viability of many strict anaerobes. While excessive exposure to oxygen could be avoided by processing samples inside an anaerobic chamber, this would add time, the standard burden associated with working in an anaerobic chamber, and the need for an anaerobic chamber dedicated to fecal processing. Furthermore, once the samples are frozen, retrieving a consistent amount of material from each tube for follow-up procedures (e.g., screen in 96-well plate) is quite cumbersome as samples stick to the tube (i.e., they do not come out), their removal require the use a small spatula (a new spatula for each sample), and in our experience by the time the operator has managed to scoop the sample out of the tube a large fraction of it has thawed, which is not desirable. Moreover, while immediate sample processing and full homogenization are preferred, these methods are not always possible in the field or for geographically disperse samples.

Here, we present the Fecal Aliquot Straw Technique (FAST), a novel fecal processing and storage method. FAST allows easy, reproducible fecal processing and storage with minimal supplies. FAST-processed samples can be efficiently stored at ultra-low temperatures for extended periods of time and easily subsampled without thawing. 16S rRNA gene profiling confirmed the ability for highly reproducible subsampling within a subject. Furthermore, transplantation of these samples into germ-free mice recovered on average ~ 80% of the donor bacterial community composition, retained donor individuality, and revealed large interpersonal variation in microbial choline metabolism.

## Results and discussion

### Fecal Aliquot Straw Technique (FAST)

**Fig. 1:**
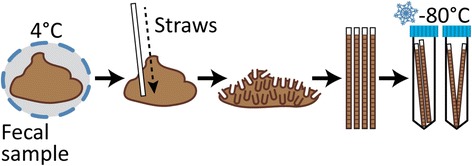
Fecal Aliquot Straw Technique (FAST). Stool samples arrived at 4 °C within 48 h of collection without evidence of freezing. Inside a biological hood, sample straws were repeatedly inserted throughout the fecal sample. This process was carried out with four straws per sample provided there was enough fecal material. Filled straws were snap-frozen and stored in sterile 15-ml tubes (2 straws/tube) at − 80 °C

Human fecal samples were collected as previously described [16]. Briefly, stool was directly defecated into a sterile container. Samples were stored and shipped at 4 °C, arriving at UW-Madison within 48 h of collection without evidence of freezing. UV-treated plastic spatulas (VWR 80081-190), that had their ends previously trimmed (i.e., straws), were repeatedly inserted vigorously throughout the fecal material in order to fill straws with a homogenized mixture and minimal air pockets. This process was carried out in a biological safety cabinet. Four straws were processed for each sample provided there was enough fecal material to do so. In the case of stool samples that were too soft or watery to fill a straw, a sterile 1 ml syringe was used. Filled straws (or syringes) were frozen on dry ice, inserted into 15 ml conical tubes (2 straws/tube), and stored at − 80 °C until later use (Fig. 1). Between samples, the operator decontaminated the working space using Trifectant and changed gloves.

### FAST enables consistent and convenient subsampling of frozen fecal material

FAST-processed samples were removed from the freezer and subsampled by slicing 1 cm sections with a sterile razor blade, while keeping the straw on a weigh boat on top of dry ice to prevent thawing. Two randomly selected straws were sampled for each subject, from here on referred to as “S1” and “S2”. S1 was used only for DNA extraction while S2 was used for preparing an oral gavage used to colonize germ-free (GF) mice (see “Methods” section). We obtained 2.5 million high-quality sequences (62,600 ± 62,700 SD per sample) of the variable 4 region of the 16S rRNA gene. This resulted in Good’s coverage > 98% for all samples (Additional file 1: Table S1) and 2723 unique 97% operational taxonomic units (OTUs) in the dataset (Additional file 2: Table S2). Rarified datasets used in alpha- and beta-diversity analyses were normalized to 4500 sequences per sample, which resulted in Good’s coverage > 98% and 809 OTUs.

**Fig. 2:**
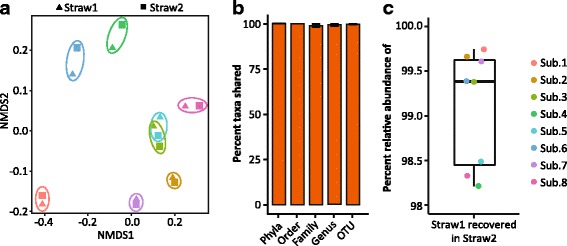
FAST subsamples capture reproducible communities. **a** Non-metric multidimensional scaling plots of the unweighted UniFrac metric between samples, colored by subject. Colored ellipses are the smallest area for human samples from each subject. **b** Taxa recovery in S2 compared to S1. Calculated with non-rarified data. Identified taxa were at least 0.1% relative abundance in at least one of the two samples in each comparison. **c** Percent relative abundance of S1 community captured in oral gavage preparation (S2). Where shown, bars represent mean ± standard error

FAST samples collected from different straws were highly reproducible. Despite the minor differences in sample processing prior to DNA extraction, the diversity and overall microbial community within subjects (i.e., S1 and S2) did not differ as assessed by the Shannon diversity index (Additional file 3: Figure S1, linear mixed effects *P* = 0.55) or unweighted UniFrac (Fig. 2a, PERMANOVA *P* = 0.75). Detection of taxa (phyla through OTU) of at least 0.1% abundance in at least one of the two aliquots sampled for each subject was nearly at 100% (Fig. 2b). For all subjects, 100% of the phyla and order-level taxa that were detected in S1 were recovered in S2. High consistency between the two aliquots was also observed at the family- (98.8 ± 0.8% SE), genus- (99.1 ± 0.6% SE), and OTU-level taxa (99.8 ± 0.2% SE; Fig. 2b). OTUs that were not recovered consistently between the two aliquots represented taxa of low abundance (0.1–0.3%). Across all samples, recovered OTUs in S2 accounted for the majority of the S1 community, summing to greater than 98% relative abundance in the S1 sample (Fig. 2c).

### Inoculation of mice using FAST-processed samples

**Fig. 3:**
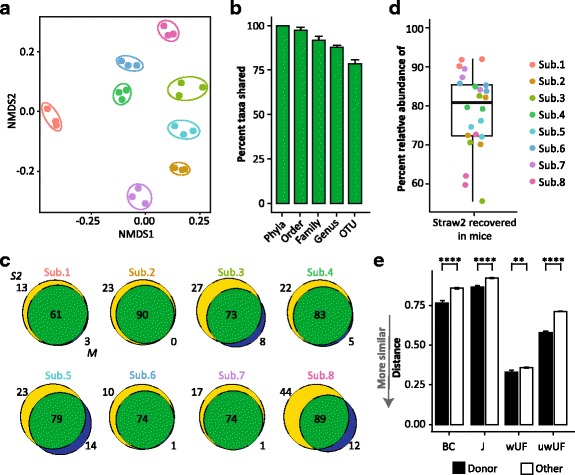
Transplanted communities capture human diversity and individuality. Germ-free B6 females were colonized with fecal slurries prepared from S2 and maintained on a chow diet for 2 weeks prior to fecal collection. **a** Non-metric multidimensional scaling plots of the unweighted UniFrac metric between mouse fecal samples, colored by donor. Colored ellipses are the smallest area for mouse-derived samples from each subject. **b** Taxa recovery in mouse fecal samples compared to oral inoculum (S2). Calculated with non-rarified data. Identified taxa were at least 0.1% relative abundance in at least one of the four samples in each comparison (S2 and 3 mouse fecal samples). **c** OTU Venn diagrams. Yellow circles represent OTUs in the oral inoculum (S2), blue circles represent OTUs recovered in the mouse (M), and green represents OTUs shared by both samples. **d** Percent relative abundance of the oral gavage community (S2) captured in mouse fecal samples. **e** Bray-Curtis (BC), Jaccard (J), weighted UniFrac (wUF), and unweighted UniFrac (uwUF) beta-diversity measures of mouse samples compared to their matched subject donor (DONOR) or compared to any non-donor subject (OTHER). ****P value < 0.0001

S2 from each human specimen was used as the oral inoculum to colonize eight separate groups of germ-free (GF) mice (3 animals/community). Colonized animals were maintained on a chow diet for 2 weeks prior to fecal sample collection. 16S rRNA gene analysis of fecal samples obtained from transplanted animals differed by subject (PERMANOVA *P* = 0.001) and clustered by donor (Fig. 3a). Recovery of phyla of at least 0.1% relative abundance in at least one of the four samples per subject (i.e., S2 and three mouse fecal pellets) was 100% (Fig. 3b). At lower taxonomic levels, 97.4 ± 1.7% of orders, 91.8 ± 2.1% of families, and 87.7 ± 1.4% genera detected in the oral inoculum were recovered in mouse feces 2 weeks after colonization (Fig. 3b). While FAST transplantation captured the majority of the human microbiota, not every abundant OTU detected in the oral gavage (S2) was successfully recovered in mice (Fig. 3b, c). OTUs recovered in the mouse 2 weeks later averaged 78.5 ± 2.2% of those present in the oral inoculum (Fig. 3b) and accounted for 78.5 ± 2.1% relative abundance of the community used as inoculum (Fig. 3d).

Transplanted bacterial communities were more similar to that of their donor than to any other human sample in the dataset (Fig. 3e, Kruskal-Wallis *P* < 0.01). However, mouse selected communities clustered distinctly from human ones (Additional file 4: Figure S2) suggesting the largest selection on bacterial taxa occurred during colonization of the mouse gut rather than in processing. Consistently in all samples, taxa belonging to the Bacteroidetes and Verrucomicrobia phyla bloomed following transplantation into mice while taxa belonging to the Firmicutes and Proteobacteria phyla decreased in relative abundance (Additional file 5: Figure S3A). Similarly, shifts in relative abundance were observed at the family (Additional file 5: Figure S3B) and genus (Additional file 5: Figure S3C) level but the direction and amplitude remained similar among subjects. There were several genera that were rarely abundant in donor samples, e.g., *Bifidobacterium*, *Collinsella*, *Dialister*, *Lachnospira*, and *Streptococcus* that failed to colonize mice. However, *Akkermansia*, *Bacteroides*, and *Sutterella,* when present in the donor sample, tended to increase in representation in mouse samples (Additional file 2: Table S2). Lost OTUs may reflect evolutionary (i.e., host adaptation), diet imparted-constraints, or result from reduced cell viability associated with the handling and freezing of the samples.

### Method application: assessing inter-individual variation in microbial choline metabolism

We used the mice mentioned above to assess inter-personal variation in microbial choline consumption and accumulation of the pro-atherogenic molecule trimethylamine-*N*-oxide (TMAO) [17]. Microbial metabolism of choline results in the production of trimethylamine (TMA), a compound that is absorbed by the host and further converted in the liver to TMAO [17]. Accumulation of TMAO thereby provides an indirect measure of microbial metabolic activity allowing us to assess functional recovery following transplantation. Colonized mice maintained on a standard chow for 2 weeks were transitioned onto a 1% (wt/wt) choline diet for an additional 2 weeks after fecal collection. Following 2 weeks on the choline-supplemented diet, serum was collected from non-fasted mice and TMAO and choline quantified using HPLC/MS.

**Fig. 4:**
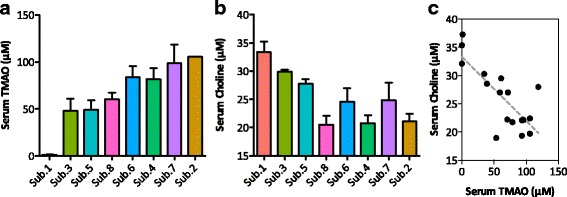
TMAO abundance inversely correlates with choline bioavailability. Colonized mice detailed in Fig. 3 were transitioned onto a defined diet containing 1% choline for 2 weeks. **a** TMAO and **b** choline were quantified from serum samples (non-fasted mice) using HPLC-MS/MS. Bars represent mean ± standard error (*n* = 2–3 mice per community). **c** Linear regression of serum TMAO vs. serum choline levels *R*^2^ = 0.5945; *P* value = 0.0003

Our results show that transplanted gut microbial communities vary in their capacity to metabolize choline (Fig. 4a). Previous studies using synthetic communities, which differ in choline utilization ability by only a single species or single bacterial gene, have shown microbial choline utilization significantly reduces the bioavailability of choline [5, 7]. Here, we show that TMAO levels are inversely associated with choline bioavailability (*R*^2^ = 0.5945; *P* value = 0.0003) (Fig. 4b, c). Interestingly, choline and TMAO level extremes measured in mice colonized with these complex human communities are comparable to those previously reported in animals colonized with synthetic (6–13 species) communities [5, 7].

Notably, mouse cohorts colonized months apart with different subsamples of an individual’s FAST samples reproducibly captured microbial metabolic activity as indicated by TMAO accumulation. For example, a non-TMA producing community (subject 1) consistently did not accumulate TMAO when transplanted into GF mice whereas a TMA-producing community (subject 8) retained TMA-production activity, as indicated by the consistently high levels of TMAO accumulation (Additional file 6: Figure S4).

## Conclusions

Changes in the specific composition (and encoded functions) of the gut microbiota have been associated with various disorders from obesity to cognitive dysfunction [9, 18]. While many of these associations still lack mechanistic details, such correlations have prompted a growing number of human longitudinal studies to incorporate human microbiome analyses into their vast metadata collections. Until now, these efforts have been largely limited to 16S rRNA gene sequencing due to both cost and processing techniques. The Fecal Aliquot Straw Technique (FAST) presented here provides an easy, inexpensive, reproducible workflow for processing and storing fecal samples (human or other animals) that can be completed in remote locations. It reduces sample volumes for shipping and enables repetitive subsampling (straw pieces) from valuable specimens. FAST minimizes risks associated with previous sampling methods [11–15, 19] including repetitive freeze-thaw cycles and overall provides a novel solution to future collection endeavors.

## Methods

### Gnotobiotic husbandry

All experiments involving mice were performed using protocols approved by the University of Wisconsin-Madison Animal Care and Use Committee. For completion of fecal transplants, female C57BL/6 (B6) germ-free mice were gavaged with ~ 200 μl of fecal inocula. Fecal suspensions were prepared under anaerobic conditions in Hungate tubes using a 1 cm piece of frozen FAST straw material and 5 ml mega media [5]. Mice were maintained on the autoclaved chow diets for 2 weeks after humanization and on the irradiated 1% Choline diet (Envigo TD.140179) for two additional weeks. Serum for TMAO and choline measurements were taken from non-fasted mice.

### DNA extraction

Samples collected for microbiota analysis included a 1 cm piece of a FAST straw (Straw1 or S1), 1 ml of the aforementioned gavage slurry (Straw2 or S2), and 3 fecal pellets collected from mice 2 weeks after humanization (mouse or M). DNA was extracted from samples according to published bead-beating procedures [3]. In short, samples were resuspended in a solution containing 500 μl of 2× extraction buffer [200 mM Tris (pH 8.0), 200 mM NaCl, 20 mM EDTA], 210 μl of 20% SDS, 500 μl phenol:chloroform:isoamyl alcohol (pH 7.9, 25:24:1), and 500 μl of 0.1-mm diameter zirconia/silica beads. Cells were mechanically disrupted using a bead beater (BioSpec Products, Barlesville, OK, USA) for 3 min at room temperature. The aqueous layer was removed and DNA precipitated using 600 μl isopropanol and 60 μl 3 M Na-acetate. Pellets were dried with ethanol and resuspended in TE. NucleoSpin Gel and PCR Clean-up Kit (Macherey-Nagel, Bethlehem, PA, USA) was used to remove contaminants. Isolated DNA was stored at − 80 °C until downstream processing.

### 16S rRNA gene V4 amplication and sequencing

PCR was performed using primers for the variable 4 (V4) region of the bacterial 16S rRNA gene [20]. PCR reactions contained 12.5 ng DNA, 10 μM each primer, 12.5 μl 2× HotStart ReadyMix (KAPA Biosystems, Wilmington, MA, USA), and water to 25 μl. Cycling conditions were 95 °C for 3 min, then 25 cycles of 95 °C for 30 s, 55 °C for 30 s, and 72 °C for 30 s, and finally 72 °C for 5 min. PCR products were purified by gel extraction from a 1% low-melt agarose gel using a ZR-96 Zymoclean Gel DNA Recovery Kit (Zymo Research, Irvine, CA, USA). Individual samples were quantified by Qubit Fluorometer (Invitrogen, Carlsbad, CA, USA) and were equimolar pooled. The pool plus 5% PhiX control DNA was sequenced with the MiSeq 2 × 250 v2 kit (Illumina, San Diego, CA, USA) using custom sequencing primers [20]. All DNA sequences have been deposited in NCBI’s Short Read Archive (PRJNA393465).

### 16S rRNA gene V4 sequence analysis

Sequences were demultiplexed on the Illumina MiSeq, and sequence clean-up was completed in mothur v.1.39.0 [21] following steps as described in [20]. Briefly, paired-end sequences were combined, and poor quality sequences were removed. The remaining sequences were aligned to the SILVA 16S rRNA gene reference alignment database [22], and very similar sequences (differences ≤2) were pre-clustered. Chimera detection and removal were performed. Sequences were grouped into 97% operational taxonomic units (OTUs) by uncorrected pairwise distances and OptiClust clustering [23]. OTUs were classified using GreenGenes [24]. Coverage was calculated using Good’s coverage.

All statistical analyses of sequence data were performed in R v3.4.0 (http://www.r-project.org/). Beta-diversity measures were calculated using data rarified to 4500 sequences per sample with vegan (Bray-Curtis and Jaccard; https://CRAN.R-project.org/package=vegan) and phyloseq (UniFrac [25]) packages in R. Differences in beta-diversity were visualized by non-metric multidimensional scaling (nMDS) plots and assessed by permutational ANOVA (vegan::adonis) stratified by subject when needed. For factors with more than 2 levels, pairwise adonis was performed with the Bonferroni correction for multiple comparisons. When group sizes differed greatly, beta-diversity was assessed using Kruskal-Wallis rank sum tests. Alpha-diversity was calculated using the rarified dataset in mothur, and differences were assessed by ANOVA or linear mixed effects models with subject as a random effect (nlme::lme; https://CRAN.R-project.org/package=nlme). Pairwise alpha-diversity tests were performed using multiple comparisons for parametric models with Tukey’s correction for multiple comparisons (multcomp::glht) [26]. All tests were assessed at significance *P* < 0.05, and values are expressed as mean ± standard error, unless otherwise noted.

Number of taxa recovered was calculated at the phylum, order, family, genus, and OTU levels using un-rarified data. For each subject, only taxa present to at least 0.1% abundance in at least one of the two samples of interest (S1 vs. S2 or S2 vs. M) were included. Percent relative abundance recovered was calculated using all OTUs present in the reference sample (S1 or S2). All code is available at https://github.com/kdillmcfarland/FAST_method.

### HPLC metabolite measurements

Serum choline and TMAO levels were measured according published methods [5]. In brief, serum samples were prepared for analysis by precipitating proteins with 4 volumes of ice-cold methanol spiked with 2.5 μM deuterium-labeled choline and deuterium-labeled TMAO internal standards. Samples were centrifuged at 18,213×*g* at 4 °C for 3 min. The recovered supernatants were diluted 1:1 in uHPLC-grade water prior to screening. After sample preparation, identification and quantitation of TMAO and choline were performed using a uHPLC (Thermo Scientific/Dionex 3000) coupled to a high-resolution mass spectrometer (Thermo Scientific Q Exactive). Liquid chromatography separation was achieved on a Dikma Bio-Bond C4 column (150 mm × 2.1 mm; 3-μm particle size) using a 7 min isocratic gradient (50:50 methanol [MeOH] − water, 5 mM ammonium formate, and 0.1% formic acid). Quantitation of TMAO (76.0762) and d9-TMAO (85.1318) was performed via targeted MS/MS in positive mode using the following fragments masses: TMAO (58.0659) and d9-TMAO (68.1301). Quantitation of choline (104.1075) and d9-choline (113.1631) was performed in positive mode with full-MS scan by monitoring their exact masses.

## Additional files

Additional file 1: **Table S1.** Sample sequence parameters. (XLSX 14 kb)

Additional file 2: **Table S2.** OTU Table. (XLSX 1328 kb)

Additional file 3: **Figure S1.** Shannon’s diversity of microbial communities for the eight sequenced human samples. S1 and S2 represent individual aliquots from two randomly selected straws for each subject. (PDF 302 kb)

Additional file 4: **Figure S2.** Unweighted UniFrac beta-diversity of FAST and mouse fecal microbiota. Non-metric multidimensional scaling plots of the unweighted UniFrac metric between all samples. Samples are colored by subject. Shapes indicate sample type. Black ellipses are standard error for human and mouse groups. (PDF 30 kb)

Additional file 5: **Figure S3.** Shifts in phyla, family, and genus level relative abundance following transplantation. Taxa relative abundance ((**A**) phyla, (**B**), family, (**C**) genus) in oral inoculum (S2) (*x*-axis) compared to corresponding mouse fecal samples (*y*-axis). Calculated with rarified data. Identified taxa were at least 0.1% relative abundance in at least one sample in the dataset. Low-abundance taxa (0.1–5%) are indicated in grey. Black line represents 1:1 relative abundance ratio. (PDF 434 kb)

Additional file 6: **Figure S4.** TMAO accumulation is consistent for mice colonized with different straw aliquots of the same sample. Two human communities stored by FAST (sub.1 and sub.8) were transplanted by gavage (G) into germ-free B6 female mice 2–3 times (G1, G2, or G3) months apart. Following 2 weeks on a standard chow diet, mice were transitioned to a defined diet containing 1% choline for 2 weeks. Serum was collected from non-fasted animals and TMAO quantified by HPLC-MS/MS. Bars represent mean ± standard error (*n* = 2–4 mice per community). (PDF 30 kb)
