# Supplementary figures and images for: Fecal Aliquot Straw Technique (FAST) allows for easy and reproducible subsampling: assessing interpersonal variation in trimethylamine-N-oxide (TMAO) accumulation
Source: Microbiome. 2018 May 18;6:91. doi: 10.1186/s40168-018-0458-8 (PMC5960144; doi:10.1186/s40168-018-0458-8)

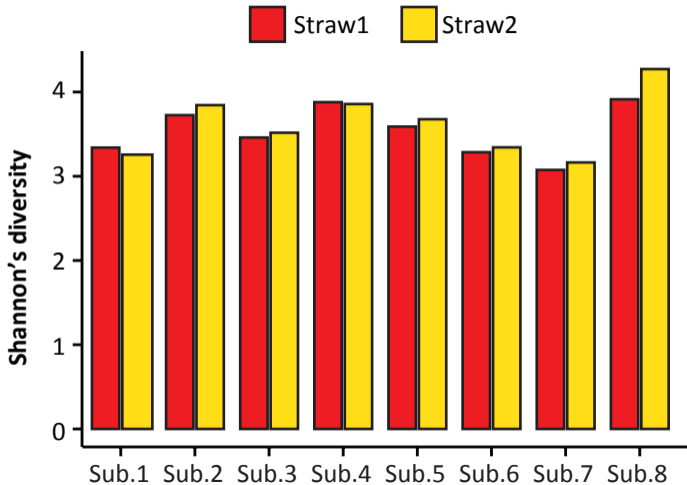

Supplement: Supplementary file 3 — Figure S1. Shannon’s diversity of microbial communities for the eight sequenced human samples. S1 and S2 represent individual aliquots from two randomly selected straws for each subject. (PDF 302 kb) [file 40168_2018_458_MOESM3_ESM.pdf]

# Unweighted UniFrac

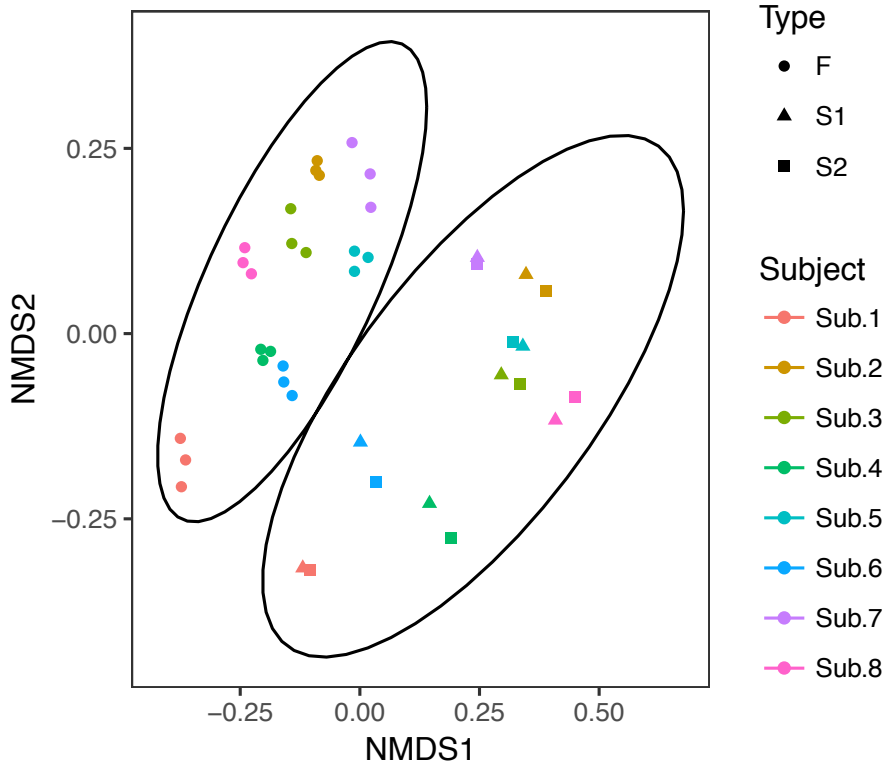

Supplement: Supplementary file 4 — Figure S2. Unweighted UniFrac beta-diversity of FAST and mouse fecal microbiota. Non-metric multidimensional scaling plots of the unweighted UniFrac metric between all samples. Samples are colored by subject. Shapes indicate sample type. Black ellipses are standard error for human and mouse groups. (PDF 30 kb) [file 40168_2018_458_MOESM4_ESM.pdf]

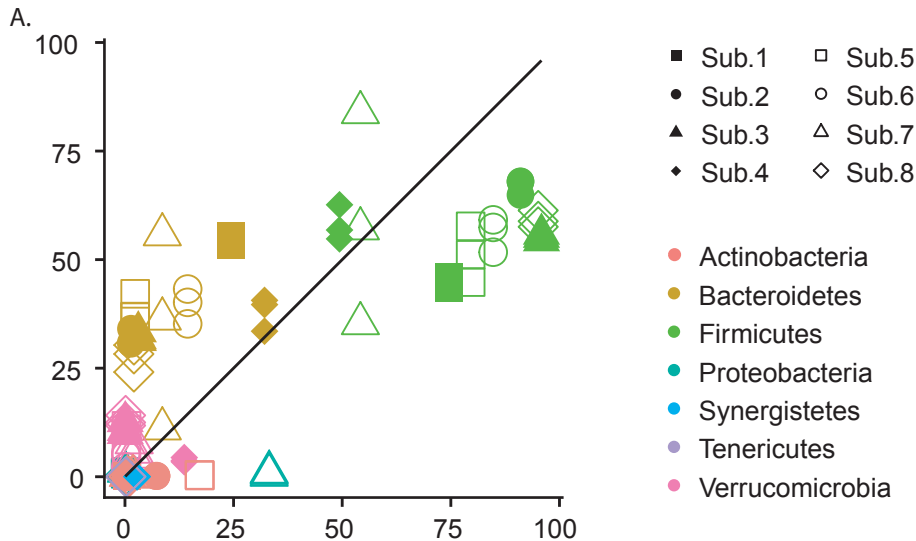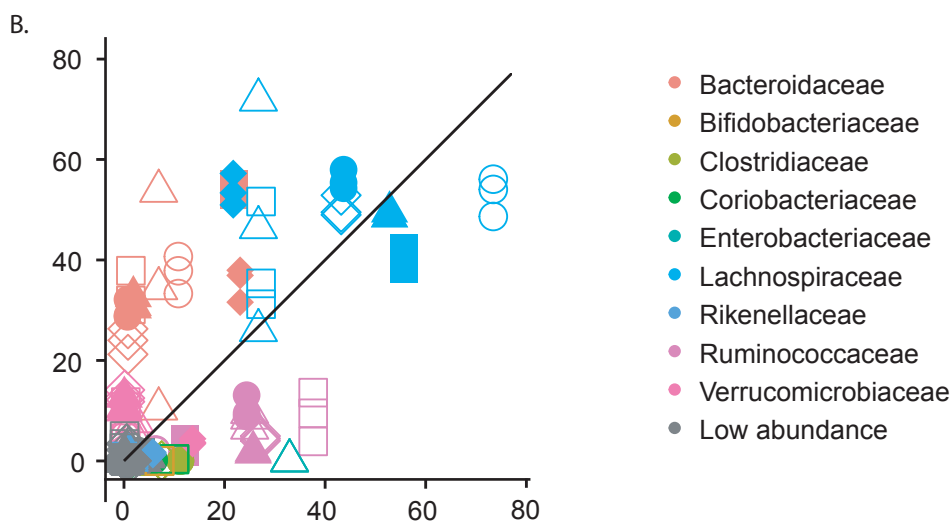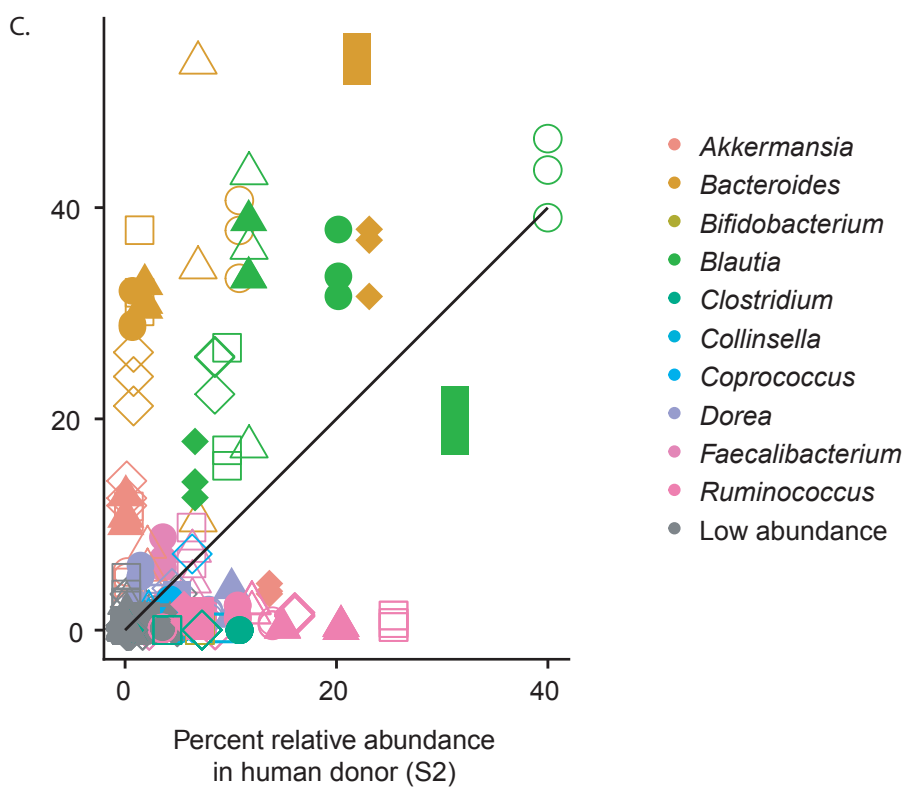

Supplement: Supplementary file 5 — Figure S3. Shifts in phyla, family, and genus level relative abundance following transplantation. Taxa relative abundance ((A) phyla, (B), family, (C) genus) in oral inoculum (S2) (x-axis) compared to corresponding mouse fecal samples (y-axis). Calculated with rarified data. Identified taxa were at least 0.1% relative abundance in at least one sample in the dataset. Low-abundance taxa (0.1–5%) are indicated in grey. Black line represents 1:1 relative abundance ratio. (PDF 434 kb) [file 40168_2018_458_MOESM5_ESM.pdf]

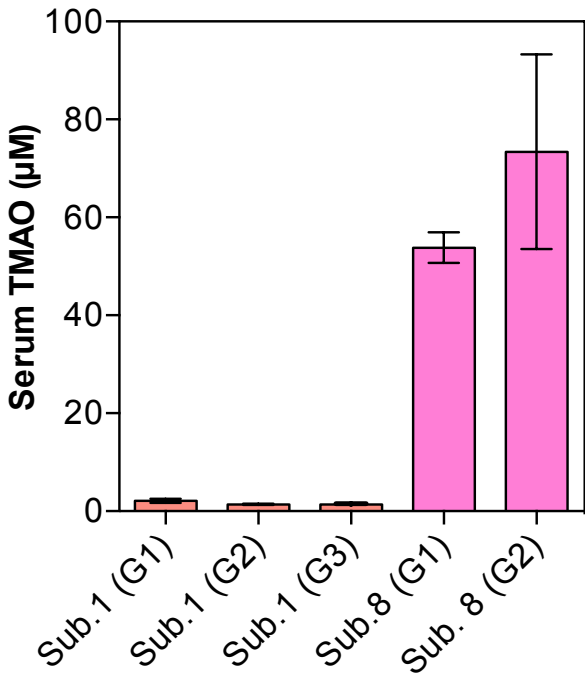

Supplement: Supplementary file 6 — Figure S4. TMAO accumulation is consistent for mice colonized with different straw aliquots of the same sample. Two human communities stored by FAST (sub.1 and sub.8) were transplanted by gavage (G) into germ-free B6 female mice 2–3 times (G1, G2, or G3) months apart. Following 2 weeks on a standard chow diet, mice were transitioned to a defined diet containing 1% choline for 2 weeks. Serum was collected from non-fasted animals and TMAO quantified by HPLC-MS/MS. Bars represent mean ± standard error (n = 2–4 mice per community). (PDF 30 kb) [file 40168_2018_458_MOESM6_ESM.pdf]
